# Neurodegenerative Diseases of the Retina and Potential for Protection and Recovery

**DOI:** 10.2174/157015908784533851

**Published:** 2008-06

**Authors:** K.-G Schmidt, H Bergert, R.H.W Funk

**Affiliations:** 1 Department of Ophthalmology, Starnberg, Josef-Jägerhuberstr. 7, D-82319 Starnberg, Germany; 2 Department of Surgery, University of Dresden, Fetscherstr. 74, D-01307 Dresden, Germany; 3 Department of Anatomy, University of Dresden, Fetscherstr. 74, D-01307 Dresden, Germany

**Keywords:** Neurodegeneration, neuroprotection, retina, glaucoma, diabetic retinopathy, age-related macular degeneration, retinal ganglion cells.

## Abstract

Recent advances in our understanding of the mechanisms in the cascade of events resulting in retinal cell death in ocular pathologies like glaucoma, diabetic retinopathy and age-related macular degeneration led to the common descriptive term of neurodegenerative diseases of the retina. The final common pathophysiologic pathway of these diseases includes a particular form of metabolic stress, resulting in an insufficient supply of nutrients to the respective target structures (optic nerve head, retina). During metabolic stress, glutamate is released initiating the death of neurones containing ionotropic glutamate (N-methyl-D-aspartat, NMDA) receptors present on ganglion cells and a specific type of amacrine cells. Experimental studies demonstrate that several drugs reduce or prevent the death of retinal neurones deficient of nutrients. These agents generally block NMDA receptors to prevent the action of glutamate or halt the subsequent pathophysiologic cycle resulting in cell death. The major causes for cell death following activation of NMDA receptors are the influx of calcium and sodium into cells, the generation of free radicals linked to the formation of advanced glycation endproducts (AGEs) and/or advanced lipoxidation endproducts (ALEs) as well as defects in the mitochondrial respiratory chain. Substances preventing these cytotoxic events are considered to be potentially neuroprotective.

## INTRODUCTION

Neuroprotection is a topic of a growing number of studies [8, 20, 23, 36, 44, 69, 96, 102, 113, 125 -128, 130 -132, 152, 158, 181] as several ocular pathologies (e.g. special forms of glaucoma [126-128], diabetic retinopathy [75, 120] and age-related macular degeneration (AMD) [107, 127] result in neurodegeneration especially of the retinal ganglion cells (RGCs). Consequently, it seems a promising goal to rescue e.g. RGCs or the retinal pigment epithelium (RPE). Indeed, a functional injury of the RGCs (which, in this respect a very vulnerable cell population) preceeds the onset of a structural damage [39, 126, 127, 130]. Therefore, a recovery would possibly restore their function. However, till now only few substances have been demonstrated to have a neuroprotective capacity and which can be pinpointed to a defined causal mechanism. In addition, such substances should be safe and easily applicable to the eye.

In this review we will concentrate on critical sites of retinal neurodegenerative diseases and possible ways of protection and ways leading to recovery. Critical sites are the RGCs (involved in the progress of glaucoma) the retinal microvessels (additionally damaged in diabetic retinopathy) and the RPE (together with vascular and RGC damages in AMD).

RGCs have a very high metabolic rate - this becomes obvious when considering that the length of an RGC axon would measure about half a mile if the cell body would have the size of an apple. These long axons increase RGC vulnerability to various disorders: during the course of their axons they are likely to encounter metabolic stress like hypoxia, exposure to increased free radicals, mechanical compression (e.g. in the lamina cribrosa - LC) and specifically photooxidative damage by (mainly blue) light passing through the retina and thus potentially damaging the retina and the retinal pigmented epithelium [171].

**Fig. (1):**
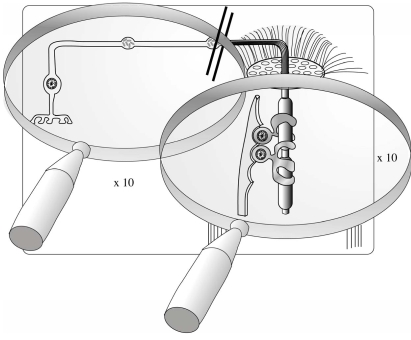
Schematic drawing of a retinal ganglion cell (RGC) axon being non-myelinated from the retina to the lamina cribrosa (LC) and being myelinated after passing this structure (Fig. **1**).

RGCs need to compensate for another unique functional situation when compared to other neurons: their axons are non-myelinated from the retina to the LC and are myelinated after passing this structure (Fig. **1**). Normally, large myelinated neurons are in the opposite situation: they lose their myelin sheets only at their very peripheral end. This special RGC feature leads to an “impedance mismatch” [192] which requires a lot of energy. Therefore, RGCs have plenty of protrusions in their axons which are filled with numerous mitochondria [192]. Furthermore, the distribution of the mitochondria (high numbers in retina, in the peripapillar and papillar zone down to the LC and much reduced in the myelinated nerve) reflects the functional requirements of different RGC axon regions [13].

Although RGCs have a high energy demand, the retinal microvasculature cannot be formed as dense as in the brain because the inner retinal layer is required to remain transparent to enable its biological function and let photons pass to the photoreceptors in the outer retinal layer. Thus, nature made a compromise between the metabolic demand of the neurons and a sufficient vascular supply [41]. Indeed, compared to capillaries in other organs, the retinal capillaries are very thin, have a high blood flow velocity, and a relative sparse network [41]. A detailed analysis of the vessel architecture reveals that on the arteriolar side of capillary network (where more oxygen is available) the mesh is wider than on the venular side (Figs. **2a**, **b**). Thus, the minimum of oxygen supply for neurons and glial cells appears to determine the vessel density in the retina.

As other big ganglion cells like the cells in the substantia nigra (which die during the development of M. Parkinson) the RGCs are relatively more prone to accumulation of metabolic end (“waste”) products which cannot be removed from the cell. In phases of increased IOP as in phases of oligemia and hypoxia followed by reperfusion, the production of free radicals and reactive oxygen species (ROS) dramatically increases [113].

As effective antioxidant capacities are generally low in most neurons and nucleic acid repair mechanisms are insufficient (especially in mitochondria) these stressors would induce RGC death. To compensate for these stressors RGCs have a high antioxidant capacity (due to endogenous peroxidases) when compared to other neurons – [85], but RGCs are still more vulnerable than e.g. Müller or vascular cells.

The same is true for the RPE which has the burden of steadily ingesting the damaged (e.g. *via* radicals by high energetic-, short wavelength light- and chemical – metabolic- “attack”) membrane disks of the photoreceptors (see below).

## FACTORS COMPROMISING RETINAL CELL FUNCTION

### Advanced Glycation Endproducts (AGEs)

Another chemical “attack” -linked with the production of radicals- is the formation of advanced glycation endproducts (AGEs) and/or advanced lipoxidation endproducts (ALEs). The accumulation of AGEs during the Maillard reaction is associated with the risk of diabetic neuropathy, diabetic retinopathy (the RGCs being the most vulnerable cell population, 44], AMD [77, 172 - 175] and M. Alzheimer [1]. Extracellular effects of the AGEs, like crosslinking of proteins, are known since the 80ties, whereas the effects of AGEs on the cellular function are still under investigation.

**Fig. (3):**
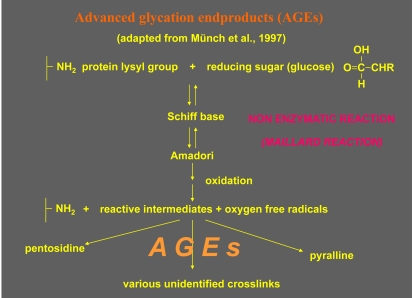
Schematic drawing indicating the reactions leading to advanced glycation endproducts (AGEs), cross-linking of protein-(lysil-residue) and reducing sugar groups (Maillard reaction). AGEs impair intracellular functional proteins and crosslink extracellular material. The time frame of the reversibility of these reactions depends on the toxicity of respective intermediary metabolic products (e.g. glyoxal).

AGEs/ALEs can form on the amino groups of proteins, lipids and DNA through a number of complex pathways including non-enzymatic glycation by glucose and reaction with metabolic intermediates and reactive dicarbonyl intermediates (Fig. **3**). These reactions not only modify the structure and function of proteins, but also cause intra-molecular and intermolecular cross-link formation. AGEs/ALEs are known to accumulate in the diabetic retina where they may have important effects on retinal vascular cell function, as determined by a growing number of *in vitro* and *in vivo* studies.

Since AGEs are constantly forming under physiological conditions, complex receptor systems have evolved to remove senescent, glycation modified molecules and/or degrade existing AGE crosslinksfrom tissues thereby limiting their deleterious effects. Suchreceptors play a critical part in AGE related biology and thepathology associated with diabetes and ageing. Several AGE binding molecules have been described and it has been established that many of the adverse effects caused by advanced glycationare mediated *via* AGE receptors such as RAGE [151] the AGE receptor complex (AGE-RC) [101, 175] and the type I and II scavenger receptor [63, 174]. Some or all AGE receptors serve to promote or limit AGE mediated cell and tissue dysfunction. AGE receptor binding can initiate important signalling pathways involving activation of protein kinase C [108, 165], tyrosine phosphorylation of Janus kinase (JAK)/signal transducers and activators of transcription (STAT) [66], recruitment of phosphotidylinositol 3' kinase to Ras, [30] and induction of oxidative stress cascades which lead toNFκB and AP-1 transcription [112, 167].

The chronic cellular activation is induced by the AGE receptor (RAGE) [9]. Sustained RAGE-mediated cellular activation has been shown to contribute to disease progression in diabetes, Alzheimer’s disease, rheumatoid arthritis, elastosis, pulmonary fibrosis, and various cancers [18, 82, 135, 180, 188] AGE and other RAGE ligands activate p21ras, MAP ERK1/2 kinases, and NFB nuclear translocation, altering expression of genes involved with cellular stress [172, 188].

### AGEs in Retinal Neurodegeneration

Like in other vascular beds AGEs and/or late Amadori products have been localised to retinal vessels and neuroglia of diabetics [15, 54, 117, 150, 175]. In diabetic rats, AGEs are not only localised to vascular basement membranes (BMs), but also appear to accumulate in the retinal pericytes after 8 months of diabetes [174]. Moreover, when non-diabetic animals are infused with preformed AGE albumin, these adducts accumulate around and within the pericytes, co-localise with AGE receptors, induce BM thickening, and cause breakdown of the inner blood-retinal barrier [25, 173, 174]. In clinical studies it has been reported that the levels of serum AGEs, and also the glycoxidation product CML, correlate with the degree of diabetic retinopathy [22, 123]. In hyperglycemic mice, AGEs lead to early inner retinal neuronal dysfunction. Here, AGEs were also localized to the vitreous cavity and internal limiting membrane (ILM) of the retina, where they were intimately associated with the footplates of RAGE-expressing Muller cells. Furthermore, AGE accumulation was increased within the retinal extracellular matrix and attenuation of the RAGE axis with soluble RAGE ameliorated neuronal dysfunction and reduced the development of capillary lesions in these mice [7].

AGE deposits were found in AMD retinas. Furthermore, AGE stimulated RAGE-mediated activation of cultured RPE cells in a dose-dependent manner. Thus, AGE accumulation may induce receptor-mediated activation of RPE/photoreceptor cells, contributing to disease progression in the aging human retinas.

AGEs have been reported to accumulate in aging eyes in Bruch’s membrane, drusen, subfoveal neovascular membranes, and RPE cells [65].

RPE cells are radically influenced by exposure to AGEs *in vitro* where they express abnormal levels of vascular endothelialgrowth factor (VEGF) and platelet derived growth factor B (PDGF-B) [56, 98]. This may have a bearing on RPE cell function, maintenance of the choriocapillaris, and integrity of the RPE/photoreceptor complex.The accumulation of lipofuscin and reduction of lysosomal degradative capacity in RPE cells may reflect AGE formation and receptor mediated transport of these adducts to the lysosomal compartment. Significantly, intracellular sequestration of these highly reactive adducts can markedly reduce lysosomal enzymatic activity in other epithelial cell types and lead to lipofuscin in RPE cells [11, 175].

Also a significant reduction of the velocity of intracellular microvesicles was induced by AGEs, the reason for this is unclear [146]. It could be due to disturbed calcium metabolism or caused by microtubuli-changes. The aggregation of intracellular microvesicles could be the result of an altered binding-behaviour of the vesicles or the destruction of the transport apparatus. The proteins dynamin 2 and clathrin, which are involved in transport apparatus, show changes in intracellular distribution indicating a breakdown of the normal cellular distribution system [146].

**Fig. (4):**
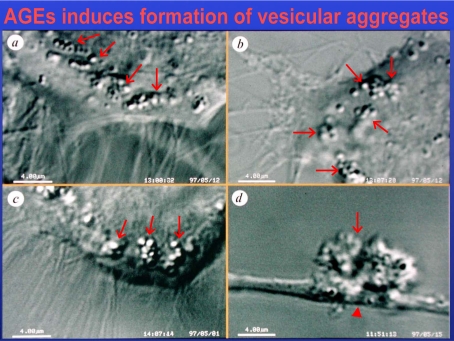
Differential interference contrast microscopy (DIC) of living astrocytes treated with glyoxal, a reactive intermediate of AGE-production. This leads to accumulation of isolated and aggregated intracellular vesicles (arrows, a-c) in the axons of a neuron and to blockade of the axonal transport (arrowheads, c,d).

In AGE-loaded RGCs, “breaks” in the tracks of axonal vesicle transport occur which subsequently lead to protrusions of the axons and to accumulation of the transported material. Possibly, AGEs may affect the axonal transport either directly by cross-linking the proteins or by enhanced production of ROS (Fig. **4**).

On the other hand all other systemic metabolic problems of the organism like diabetes mellitus, hyperlipidemia, hypercholesterolemia, hyperuricemia, high systemic blood pressure and periods of very low perfusion pressure due to vasospasm also affect the microenvironment of the RGCs and RPE – from the capillaries *via* the extracellular space *via* the glial cells down to the intracellular “milieu” of these neurons. Furthermore, the above mentioned metabolic stressors often combine (“metabolic syndrome”) and can damage the RGC microenvironment *via* thickening of the basement membranes, e.g. due to AGEs/ALEs. In this respect it is of considerable interest that some types of M. Alzheimer’s disease (AD) are very similar to dementia caused by arteriosclerosis [186]. Here, it is also unclear why many diabetic damages leave the glia cells of the brain unaffected (despite AGE and lipid peroxide formation) for a long time whereas in certain forms of arteriosclerosis the glial cells become “aggressive” and attack the nerve cells and axons with ROS (e.g. as in “white matter lesions” of the brain).

A study in a mouse model showed that the combination of deleterious factors such as apolipoprotein (APOE) allelic expression, advanced age and diet contribute to atrophic degenerative changes in RGCs and the optic nerve [191]. Another study, also in an animal model demonstrated that the cleavage product of the amyloid precursor protein (APP) (major indicator of AD) is enhanced in ocular hypertension [47] and axonal damage. High IOP, induced hypoxia, lipid peroxides and AGEs *per se* lead to increased production of free radical species which again initiate periods of cell damage and attempts to regenerate parts of the cell, especially the RGC - axon. Here, the pathogenetic processes in the RGCs are analogue to those in brain neurons during AD:

The repeated stimuli for regeneration of RGCs and neurons in general are characterized by the production of an enormous amount of newly synthesized proteins (like APP) needed for the realignment and restoration of the axon. Therefore, the cell tries to transport all proteins needed to the (peripheral) damaged area (e.g. sAPP which is required during migration and proliferation – 138; and APP as membrane protein which binds kinesin to vesicles which are transported with the fast axonal transport, 74, 166).

On the other hand, APP plays an important physiological role in protecting neurons from the consequences of prolonged endoplasmic reticulum stress which is found particularly in AD [83]. Furthermore, the microtubule associated protein tau inhibits kinesin-dependent transport of peroxisomes, neurofilaments, and Golgi-derived vesicles into neurites (axons). Loss of peroxisomes on the other hand, makes cells vulnerable to oxidative stress and this leads to degeneration. Again, tau inhibits the transport of APP into axons and dendrites, causing its accumulation inthe cell and thus initiates apoptosis - also mediated by caspases 8 and 3 [106, 187]. These factors facilitate degenerative processes as mentioned above.

## RADICALS IN OXIDATIVE STRESS AND RPE DYSFUNCTION

Although oxidative stress and RPE dysfunction are generally believed to promote disease progression in AMD, the underlying mechanisms governing these events are poorly understood. The inherently high arterial O_2_ tension environment, production of radicals in phototransduction, blue light damage, accumulation of photooxidative lipofuscin containing A2E in the RPE, and loss of cellular antioxidant capabilities collectively contribute to oxidative stress in the aging eye [10, 103, 171, 177, 195]. Correspondingly, RPE and choroidal cells alter the expression of genes for cytokines, matrix organization, cell adhesion, and apoptosis [2, 34, 48, 49, 53, 61, 116, 183]. Chronic cellular activation perturbs normal structural and physiological integrity and may induce focal inflammatory responses at the RPE–Bruch’s membrane border [65].

Furthermore, the formation of AGE, such as CML and pentosidine, is accelerated in regions of oxidative stress.

Recent papers also report ROS attack of neurons and a loss of antioxidant capacity within the neurons in the course of some forms of glaucoma *via* aggressive glial cells [122]. In addition, ROS, induced e.g. by hypoxia are further increased in the presence of AGEs [112].

## CELL STRESS AND APOPTOSIS

**Fig. (5):**
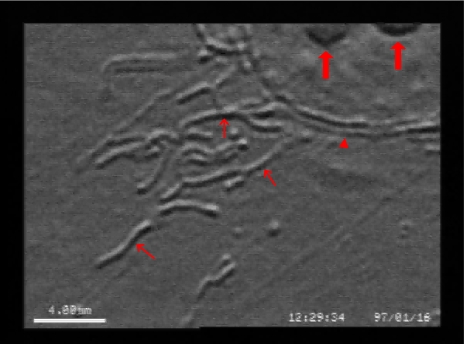
Differential interference contrast microscopy (DIC) of a cultured astrocyte, the mitochondria are clearly outlined (thin arrows). Arrowhead: membrane of the nucleus, thick arrows: nucleoli.

**Fig. (6):**
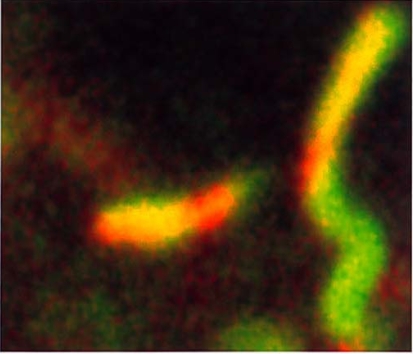
Cross section of single mitochondria (width: 0.5 µm) in an astrocyte: the function of the mitochondrial membrane is clearly indicated by the colour of a dye (JC1) indicating the degree of the membrane potential: red still intact; green compromised.

For early detection of cell stress preceding apoptosis our own group monitored the course of the alteration of pHi and mitochondrial membrane potential in a retinal ganglion cell line [80]. Changes in pH are early events in the progression of cell stress long before the way to apoptosis is irreversible. The efficiency of activation of caspase by cytochrome c has been found to be pH sensitive, with a pH optimum of 6.3-6.8 *in vitro* [70]. Alterations in cytosolic pHi may be caused by changes in mitochondria (Fig. **5**), such as the deleterious opening of the mitochondrial permeability transition pore. Mitochondrial permeability transition is a non-selective increase in the permeability of the inner membrane (presumably involving a multi-protein complex known as the permeability transition pore whose opening commonly occurs during apoptosis) and results in depolarization of mitochondria and loss of the H^+^ gradient normally present across the inner membrane (Fig. **6**). Non-selective entry of ions and water into the solute-rich matrix then leads to an increase in the volume of the mitochondrium [42]. Caspase activation can be a result of mitochondrial-matrix alkalinization and cytosolic acidification [105]. Cytochrome c, normally stored between the inner and outer membranes of mitochondria, is commonly released into the cytosol following exposure of cells to apoptotic stimuli. Once it is in the cytosol, cytochrome c binds to the caspase-activating protein Apaf-1, inducing formation of an oligomeric complex that recruits and proteolytically activates procaspase-9, an activated caspase-9 that then cleaves and activates caspases further downstream, ultimately inducing apoptosis [92].

Mittag *et al*. 2000 [111] showed that the mitochondrial membrane potential is a good marker for apoptosis of retinal cells *in vivo* after intravitreal injection of an indicator dye. In the retinal ganglion cell layer of eyes with elevated pressure, mitochondrial membrane potential was reduced by 17.5%. After 3.5 months of elevated IOP the retinas showed cell nuclei at various stages of apoptosis, from the initial DNA condensation to fragmentation.

If glutamate (the major excitatory transmitter in the retina) accumulates as a further consequence of cell stress, uptake of cystine is inhibited which is essential for glutathione (GSH, the most important intracellular antioxidant) biosynthesis, resulting in a depletion of GSH from the cells. This, again, causes an increase of ROS levels [100] which leads to a Ca2+ influx which is mediated by a cobalt sensitive, cyclic guanosine monophosphate (cGMP) – gated Ca2+ channel [92].

All the processes described above are able to reduce cell functions and can lead to apoptosis. In this respect a special form of prolonged apoptosis has recently been found in neurons of the brain. In the RGCs this prolonged apoptosis can last for many years [206].

### Summary of the Pathogenetic Processes

For the mentioned neurodegenerative diseases (special forms of glaucoma, diabetic retinopathy and AMD) many findings converge to an age – and metabolic disorder - dependent damage of cellular energy - (mitochondria) and transport processes (e.g. endo – lysosomal pathway and axonal transport). These damages are mostly caused by ROS and all other processes which are linked to radical production: AGEs, ALEs and oxysteroles. A changed metabolic situation can also lead to aggressive glial cells which by themselves produce radicals which vice versa crosslink proteins and lipids to AGEs and ALEs.

**Fig. (7):**
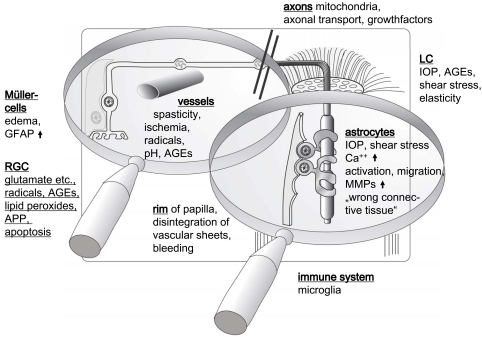
Schematic drawing and localization of the factors leading to neurodegenerative diseases in the retina (GFAP = glial fibrillary protein, APP = amyloid precursor protein, MMPs = matrix metallo-proteinases). Please see text for details.

The other hot spot in the pathomechanisms of neuodegenerative diseases (like also AD and PD) is the mitochondrium. Defects in the respiratory chain (like in LHON) are similar in age – and metabolic disorders (electrons deviate from the respiratory chain and represent additional radicals). In addition, other factors like AGEs in the endfeet of Müller cells accumulate and damage the mitochondria which are not only in the cell bodies but also in protrusions of the RGC nerve fibers (Fig. **7**) [192].

### Therapeutic Strategies

Such strategies should focus on the above mentioned "hot spots" and restore or provide the following features by amelioration of the following parameters:

- microcirculation
- route from the capillary to the cell and back
- metabolic situation within the cell
- balance of oxidative and antioxidative parameters
- Reduction of AGEs/ALEs and ROS

Prevention or amelioration of AGE mediated cell toxicity has been a key strategy in the prevention of diabetic complicationsand some age related pathology. To date there have been a range of approaches which seek to either prevent AGE formation, reduceAGE effects on cells, or even break established AGE crosslinks.

Amadori product formation is the basis of advanced glycation biochemistry because progression to protein crosslinks requires slow chemical rearrangement to create reactive intermediates before the formation of irreversible AGEs. An important pharmacological strategy for the inhibition of this process utilises the small nucleophilic hydrazine compound aminoguanidine, which is a potent inhibitor of AGE mediated crosslinking [15]. This drug can prevent some diabetic vascular complications in experimental animals, [24, 55, 133] while clinical trials of aminoguanidine were shown to effectively reduce AGE-Hb while leaving HbA_1c_ unaffected [17]. Such optimism has been tempered by the gradual realisation that aminoguanidine also inhibits a range of other important pathways, most notably generation of nitric oxide by eNOS, [72] which may increase non-specific and unwanted side effects of the drug. Other AGE inhibiting drugs have been recently developed, such as the thiazolidine derivative OPB-9195, [120] pyridoxamine, [124] and 2,3 diaminophenazine (2,3 DAP) [170].

Prevention of AGEs interacting with their receptors or other body proteins is a valid therapeutic approach. The use of neutralising antibodies against glycated albumin has been shown to prevent BM thickening in diabetic (db/db) mice despite the fact that the antibodies did not alter the glycaemic status of the animals [25]. Likewise, the use of the AGE binding properties of lysozyme has succeeded in reducing AGE levels in dialysate from diabetic patients with kidney disease [110] and presents a real possibility for reduction of toxic AGE groups in the body fluids of patients with renal failure. Furthermore, elucidation of AGE receptor signal transduction pathways may also offer intracellular strategies to control receptor mediated sequelae.

In the experimental animal, AGE effects could be significantly reversed by the pharmacological AGE inhibitor aminoguanidine [189].

Recently, a novel therapeutic strategy has been to attack the AGE crosslinks formed in biological systems. This is an exciting approach since it would "break" pre-accumulated AGEs and subsequentlyallow clearance *via* the kidney. Such an AGE crosslink "breaker" prototype has been described to attack dicarbonyl derived crosslinks *in vitro* [185]. There are now at least two such chemical agents which have the ability to reduce the tissue content of AGEs inexperimental diabetes, [26, 199] reverse hyperglycaemia related arterial distensibility, [120] and ameliorate age related myocardial stiffness [6].

## REDUCTION OF APOPTOSIS BY STABILIZING THE INTRACELLULAR PH *VIA* CARBONIC ANHYDRASE BLOCKERS 

While testing the vasodilating capacity of carbonic anhydrase blockers in *post vivo* whole mounts of the rat retina (the cells were held viable in a special observation chamber [80, 164], the group of Funk [80] found a surprising phenomenon: in the extracellular space (adjoining to the pericytes or smooth muscle cells) a decrease of the pH took place (leading also to vasodilation). However, the intracellular pH (pH_i_) of the neurons in the retinal whole mounts remained at higher levels in the carbonic anhydrase blocker treated cells compared to the untreated retina cells. This observation was the starting point to the idea that holding the pHi at normal levels would have an anti – apoptotic effect. Due to these results carbonic anhydrase blockers should then have anti – apoptotic properties.: it is known that many cell types (including neurons possess the enzyme carbonic anhydrase and on the other hand, apoptosis is often associated with decreased cytosolic pH. In neurons ischemia or oxidative stress leads to decreased pHi and this renders the cell susceptible to further damage [201, 203].

Reduced pH_i_ promotes apoptosis by favouring caspase activation (pH optimum for caspase-3 is 6.6 – 6.8, 39] and activation of DNase II, [62] but there is a controversial discussion whether low pH leads to enhanced production of free radicals (reactive oxygen species, ROS) [164] or vice versa [107]. There are also studies where acidification of cytosol was inhibited without altering the apoptotic response [44, 126]. Taken these facts together, we conclude that decreased cytosolic pH is permissive of apoptosis but until now it is uncertain whether it plays a role in signalling cell death.

In retinal neurons, advanced glycation endproducts (AGEs) [1, 145] as well as hydrogen peroxide [137] lead to acidification of the cytoplasm, to elevated ROS production and finally to apoptotic cell death [68].

### Carbonic Anhydrase Blocker Dorzolamide

We studied E1A-NR3 cells which were incubated with varying concentrations of glyoxal, methylglyoxal and H_2_O_2_ for different periods of time. To a fraction of the assays dorzolamide was added. Apoptotic changes were determined by measuring cell fluorescence with a cytofluorimeter after incubating the cells with appropriate dyes and antibodies. The following parameters were studied: DNA strand breaks (TUNEL assay), subdiploid DNA content (sub-G1 assay), binding of annexinV, production of reactive oxygen intermediates (ROS), active caspase-3, the glycation product N_є_ - (carboxymethyl)lysine (CML) and intracellular pH. Dorzolamide proved to reduce the damage, which was inflicted on retinal ganglion cells by agents that induce apoptosis and therefore this carbonic anhydrase blocker can be considered a neuroprotectant as this effect was independent of its IOP-lowering and its positive effect on ocular perfusion [163].

As mentioned above, defects in mitochondrial energy metabolism due to respiratory chain disorders lead to a decrease in mitochondrial membrane potential and induce apoptosis. Since coenzyme Q_{10} (CoQ_{10}) plays a dual role as an antioxidant and bioenergetic agent in the respiratory chain, it has attracted increasing attention concerning the prevention of apoptosis in mitochondrial diseases. In cell studies with rotenone as stressor, pre-treatment with CoQ_{10} (10 or 100 wM) for 48h led to a significant reduction of rotenone-induced loss of mitochondrial membrane potential [109]. These results suggest, that cytoprotection by CoQ_{10} may be mediated by raising cellular resistance against the initiating steps of apoptosis [109].

## NEUROPROTECTION IN PRIMARY OPEN ANGLE GLAUCOMA

Studies from POAG patients with IOPs in the normal range (normal tension POAG) demonstrated localized insufficiency in the ocular vasculature [14, 38, 40, 41, 43, 52, 59, 79, 153-157, 160-163]. Vascular risk factors (e.g. vasospasm) and absolutely (high tension POAG) and relatively (normal tension POAG) elevated IOP seem to be connected [38]. An insufficient regulation of blood flow to the optic nerve head in response to IOP compressing the microvasculature appears to increase the vulnerability of RGCs and glial cells and thus increase the risk for POAG while a sufficiently (auto)regulated optic nerve head blood flow may compensate for an increase in IOP and prevent oligemic RGC damage as e.g. in ocular hypertensive patients [156, 160, 163].

Some degree of vascular insufficiency may be tolerated by RGCs but if metabolic stress is added e.g. by increased IOP or reduced systemic perfusion (reduced blood pressure, blood loss), glaucomatous pathology may result (as in high tension POAG), whereas insufficient vascular (auto)regulation may be pathologic even with a “normal” IOP (as in normal tension POAG).

Failure to autoregulate against stressors (e.g. reduced blood pressure, increased IOP) in the vessels supplying the optic nerve head (e.g. in the perioptic short posterior ciliary arteries) or the optic nerve head microvasculature itself (compression theory) could lead to hypoperfusion and focal oligemia initiating autoregulatory mechanisms attempting to restore metabolic homeostasis, a mechanism which could result in repeated transient episodes of oligemia and - over an extended period of time - cause the gradual focal RGC loss of and thinning of the nerve fiber layer seen in POAG patients.

The evidence for a vascular pathology in POAG [14, 38, 40, 41, 43, 52, 59, 79, 153 - 157, 160-163] led to the use of ischemia/reperfusion models to investigate drugs for neuroprotective properties [23, 112, 125, 127, 130-132, 152, 158, 198].

Ischemia/reperfusion results in an energy deficit reflected in a lack of adenosine triphosphate (ATP) in mitochondria, required to satisfy the high energy demand for nerve conduction in unmyelinated neurons [99].

This reduced bioenergetic state is further compromised by an increase in glutamate, the major neurotransmitter for RGCs, to levels toxic to these neurons [23, 112, 125-132, 152, 158, 198]. The prolonged activation of glutamate receptor–coupled ion channels causes prolonged depolarization of the cell *via* Na^+^ influx and K^+^ efflux through the open glutamate receptor-coupled channels, as well as excessive Ca^2+^ influx into the cell. Among the glutamate receptors, the NMDA and kainate subtypes have increased glutamate affinity, the highest Na^+^ and Ca^2+^ conductance, and prolonged open channel times. Each NMDA/kainate receptor has four to five subunits and multiple sites for binding of further ligands. Among these are accessory binding sites for glycine or d-serine acting as a coagonist, a modulatory site binding polyamines and binding sites within the channel for drugs such as MK801 or memantine. Besides these ligand-binding sites, the receptor/channel activity is controlled by cations (Mg^2+^, Zn^2+^), by the redox state or nitrosylation of sulfhydryl groups in proteins composing the channel and by phosphorylation/dephosphorylation sites regulated by protein kinase/ phosphoprotein phosphatase enzymes [112].

Progressively accumulating evidence suggests that RGC damage in POAG occurs primarily at the lamina cribrosa reflected in structural alterations due relatively or absolutely elevated IOP which seems to reduce axonal flow and compress the optic nerve head microvessels, reducing a potentially compromised perfusion in this area even further and thus leading to oligemia, a process reducing cellular homeostasis of unmyelinated nerve fibers directly or by causing stress to astrocytes and/or oligodendrocytes resulting in liberation of toxic mediators, e.g. nitric oxide [122].

As a consequence the axonal flow from and to the RGCs and their target neurons in the brain is compromised. A reduction in axoplasmic flow of neurotrophins to RGC somata appears to be a critical step in initiating the cascade of events resulting in apoptosis, the primary cell death mechanism in POAG.

The ischemia-reperfusion, optic nerve cut or crush models are based on these hypotheses, potential target sites for neuroprotective agents acting at the level of the lamina cribrosa could be RGC axons or glial cells (astrocytes, oligodendrocytes).

Based on the etiologic concepts for POAG discussed before, any therapy for POAG protecting RGCs from death, preventing or delaying this process and drugs which save already compromised neurons or which induce regrowth of axonal/dendritic connections and restore function may be termed neuroprotective.

To avoid confusion when discussing neuroprotection in POAG it may be worthwhile to differentiate indirect neuroprotection (reducing risk factors, e.g. reducing IOP, increasing perfusion) from direct neuroprotection, a direct interaction with retinal structures preventing RGC damage.

A direct neuroprotectant would need to reach the retina to exert its pharmacologic effect which can only be proven if this drug is applied directly to the retina, an approach which is currently limited to cell culture and animal experiments (e.g. injection into the vitreous). A neuroprotective effect of a drug applied locally or systemically may be indirect due to an effect on e.g. IOP or perfusion.

A direct neuroprotective substance may reduce upregulated stimulation of ionotrophic receptors e.g. by glutamate, aspartate, NMDA, kainic or domoic acids (excitotoxicity), reduce energy deficieny (lack of ATP), maintain cell membrane ionic balance or axonal function or stop one or several of these cytotoxic processes which reinforce each other [23, 112, 125-152, 152, 158, 198].

Neuroprotective concepts for POAG aim at protecting RGCs from the initiating, extracellular cell death signals, reduce increased Na^+^ and Ca^2+^ influx into the cell directly or to block specific NMDA receptors, and on agents which block the intracellular signal cascade for RGC apoptosis (e.g. inhibition of excessive free radical formation) [44, 112, 126, 127, 132, 152, 158].

### Na^+^ and Ca^2+^ 

In ischemia-/reperfusion damage Ca^2+^ overload is preceeded by an intracellular accumulation of Na^+^ as a result of a blockade of the Na^+^/K^+^-exchange, an increase in the Na^+^/H^+^ exchange and the Na^+^-HCO_3_ cotransport due to lack of ATP and acidosis and the reversal of Na^+^/Ca^2+^ exchange. Increased intracellular Ca^2+^ levels impede the various messenger functions of this ion and lead to a liberation of transmitters, which activate certain receptors (e.g. ionotrophic glutamate receptors) and thus further increase intracellular calcium. Na^+^ and Ca^2+^ can increase Ca^2+^ influx into the cell by activating voltage dependent canals. As a result a further increase in intracellular Ca^2+^ will disturb cellular homeostasis e.g. by activating cytotoxic enzymes, apoptosis is initiated.

Ca^2+^ overload and metabolic stress caused by glutamate excitotoxicity e.g. due to ischemia trigger production of further toxic mediators (e.g. nitric oxide - NO, free oxygen radicals) and activate mitochondrial apoptotic signal transduction pathways. Ca^2+^ acts as a cosignal with PSD-95, a postsynaptic density protein for activation of neuronal NOS and thus couples NMDA receptor activation to NO, which, in excess, is toxic to RGCs [149]. Thus, substances which reduce Na^+^ and/or Ca^2+^ influx into the cell in pathologic conditions (i.e. in persistent depolarization) may reduce or even halt cell death.

Several clinically well known drugs e.g. lidocaine, flunarizine, diazepam, betaxolol carbamazepine and phenytoin are Na^+^ channel blockers and significantly reduce cell damage [112, 126, 127, 152, 158, 198]. Carbamazepine and phenytoin used to treat epilepsy also reduce hypoxic damage in rat optic nerve culture [37]. Ca^2+^ channel blockers, such as flunarizine [35] and nifidipine [27], vasodilators used in systemic antihypertensive therapy, demonstrated a cytoprotective effect in retinal cells after ischemia/reperfusion.

Furthermore calcium channel blockers (e.g. nimodipine, nifidipine) seem to have a positive effect on the visual field prognosis in normal tension POAG [28, 78, 161].

It is uncertain whether this clinical effect is a result of direct cytoprotection or due to improved microcirculationto the optic nerve head[153, 161, 162] or a combination of both effects.

Following this concept, verapamil, a combined Ca^2+^ and Na^+^ channel blocker may have greater neuroprotective potency.

Further studies on the various neural Ca^2+^ and Na^+^ channel subtypes and the (state-dependent) binding at multiple sites of potential blockers may help to find channel type(s) specific for POAG and the drug(s) to selectively block these channels to a degree providing neuroprotection for POAG patients without compromising cell function.

## THE N-METHYL-D-ASPARTAT (NMDA)-RECEPTOR

Elevated IOP in POAG patients is associated with increased glutamate levels [31], a similar increase of this neurotransmitter was found in the vitreous of rabbits following retinal ischaemia [76] and in the aqueous humour following optic nerve crush in rats [204].

This mechanism seems to be similar in other neural tissues, e.g. the central nervous system where primary ischemic damage is also associated with glutamate induced cell death.

Prolonged NMDA - receptor overactivation by increased levels of glutamate (excitotoxicity) due to e.g. increased IOP and/or oligemia [112, 127, 130, 131, 152, 158], leads to depolarisation - which in excess - initiates Na^+^ and Ca^2+^ influx into the cell, the first step in the cascade of events resulting in apoptosis.

So it seems conclusive that NMDA antagonists may delay or halt ganglion cell death. Respective studies demonstrate that NMDA antagonists such as MK801, ifenprodil, and memantine reduce the loss of RGCs in optic nerve crush, ischemia/reperfusion, and chronic high IOP experiments [46, 152, 204].

However it remains unclear whether the small but significant increase of glutamate in the vitreous of POAG patients reflects toxic concentrations of this neurotransmitter at the level of RGCs or whether this is a compensatory mechanism of the glutamate/glutamine metabolism in the process of maintaining normal visual function.

While there is some evidence for the potential role of glutamate in the pathogenesis of POAG, its metabolism needs further investigation.

An excitotoxic increase in glutamate in the extracellular space initiates the death of neurones that express ionotropic glutamate (NMDA) receptors [12, 136], e.g. ganglion cells and a subtype of amacrine cells.

Glutamate could be released from retinal neurones as a reaction to ischemia or arise from a malfunction in the uptake/turnover of this neurotransmitter e.g. in Müller cells.

Glutamate uptake and metabolizing glutamate to glutamine is an energy (ATP) consuming process. ATP is provided by glycolysis. Thus, any impairment of glucose supply or metabolism that reduces ATP in Müller cells, such as oligemia, may reduce glutamate uptake from the extracellular space, thus increase extracellular level of this neurotransmitter, which reduces cellular homeostasis and increases the possibility of RGC apoptosis.

It is clear from a variety of studies that not all RGCs are equally sensitive to a change in cellular homeostasis. As discussed before, several studies suggest that M cells are more sensitive to destruction in POAG than P cells [139, 140], which is in keeping with the finding that the latter ganglion cells are less sensitive to intraocular injection of NMDA or glutamate than M cells [32] and thus supports a role for glutamate in glaucomatous pathology. The variable sensitivity may be explained by the variable profile of excitatory (e.g. glutamate – depolarisation) and inhibitory (e.g. aminobutyric acid, GABA – hyperpolarisation) receptors expressed by a specific subset of RGCs.

Thus the degree of depolarisation (i.e. susceptibility to apoptosis) of any GC is determined by the ratio of excitatory *vs*. inhibitory receptors expressed by that cell, i.e. a cell that expresses more GABA receptors than glutamate receptors should be more resistant to excitatory damage than a cell with fewer GABA receptors.

The receptor profile of ganglion cells is not limited to GABA and glutamate receptors. Ganglion cell function is further influenced by nicotinic [176], adenosine [90] and α_2_-adrenergic [73], both hyperpolarizing receptors.

A direct blockade of excitatory receptors to halt ganglion cell death in POAG seems an obvious therapeutic option.

Systemic [88] or intraocular [193] administration of MK-801, an NMDA receptor antagonist can protect against many of the destructive effects of either NMDA or experimental retinal ischaemia in RGCs. Memantine, another NMDA receptor antagonist demonstrated a neuroprotective effect in RGCs when exposed to glutamate in a concentration otherwise toxic to theses cells [190].

Currently, clinical use of NMDA blockers is limited by their side effect profile. As NMDA receptors are widely distributed in the central nervous system and involved in a number of vital functions, a large spectrum of side effects can be expected. E.g. patients who received MK-801 for stroke treatment showed neurotoxic reactions [84].

Interestingly, memantine acts similar to MK-801 but does not display the side effects noted with MK-801 [184], a low affinity for the NMDA receptor and a membrane stabilising effect [134] may explain this effect.

Studies on NMDA receptor subtypes and their specific properties e.g. their ability to influence the activity of a receptor by allosteric change will help to find antagonists for specific NMDA receptors and may increase neuroprotective potency for a specific neurodegenerative disease (e.g. stroke, M. Alzheimer, POAG) and reduce (neuro)toxic side effects. This is crucial for any therapy of POAG, where drugs have to be applied prophylactically for life.

## FREE RADICALS 

Free radicals are formed as part of cellular processes [71]. Oxygen and free oxygen radicals can damage most macromolecular cellular structures, as a consequence an alteration in proteins, lipid peroxidation and destruction of nucleic acids may result [71].

Certain stress conditions (hypoxia, ischemia/reperfusion) lead to excessive free oxygen radical formation which increases e.g. p53 expression neurons [147].

Upregulation of p53 increases expression glyceraldehyde-3-phosphate dehydrogenase (GAPDH), which is the major source of NADH for mitochondrial oxidative phosphorylation. An increase in the cytoplasmic NAD^+^/NADH ratio liberates the bound form of this proenzyme from its RNA binding site to active GAPDH.

SH oxidation or nitrosylation by NO also free GAPDH, but this process also inactivates GAPDH. Thus GAPDH inhibition by free oxygen radicals leads to a reduction in intracellular NADH and thus may induce apoptosis.

The finding that mice deficient of p53 are resistant to excitotoxic neuronal damage [115] is consistent with this concept.

Cellular protective mechanisms can counteract this damage with the support of specific enzymes (e.g. glutathione peroxidase, glutathione reductase, catalase, superoxid dismutases) and antioxidants (e.g. α-tocopherol, ascorbic acid, glutathion) seem to prevent apoptosis under certain conditions [71].

While the ethiopathogenetic mechanisms of POAG remain unclear it is rather obvious that cell death in this form of progressive optic neuropathy occurs by apoptosis as well as by necrosis, in other words: apoptosis and necrosis could be different forms of the same death process determined by the degree of insult.

Free oxygen radicals and respective free radical scavengers (antioxidants) seem to affect cell death in POAG independent of its mechanism. This oxidative stress may be induced by an elevation of nitric oxide due to oligemia eventually causing oxidative/nitrosative stress and lipid peroxidation of retinal ganglion cells [158]. Studies in rabbits show increased formation of free oxygen radicals after ischemia [119] and reduced retinal function, an effect which could be reversed by antioxidants [119].

Consequently antioxidative capactivity, a marker of free radical formation is increased in the aqueous humour of initial POAG but not in a later stage, which may reflect a local mechanism to compensate for increased oxidative stress in this optic neuropathy exhausted with progression [159].

Also a cytoprotective effect of various antioxidants (vitamin E, 178), katalase [186] and ginkgo biloba [178] was shown in retinal cells following ischemia/reperfusion. Lipidperoxidation, a consequence of free radical formations was reduced by tririlazadmesylate, RGC death due to lack of ATP was reduced in cell culture experiments [91].Osborne *et al*. [129] demonstrated that light damages isolated mitochondria which correlated to exposure and that light triggers apoptosis of cultured RGCs, an effect exacerbated in nutritionally deprived cells and thus propose that reactive oxygen intermediates (ROI) generated in RGC axon mitochondria due to light (especially short blue wave light - (450 – 490 nm) exposure may further compromise the survival of these neurons in POAG, where RGCs are in an bioenergetically low state (as discussed in detail before) and thus their ability to scavenge ROI is reduced, resulting in toxic ROI concentrations accelerating RGC death in POAG and mitochondrial optic neuropathies.

Antioxidants which counteract cell death independent of the exact mechanism (apoptosis or necrosis) may prove beneficial in the treatment of POAG.

## NEUROPROTECTIVE TREATMENT OF POAG

The ideal POAG drug is well tolerated orally, targets specific receptors of specific RGC subpopulations (e.g. M cells) or glial cells to reduce side effects and prevents cell death independent of the cell death mechanism.

As currently there are no drugs available which meet these criteria even in part, considerable side effects limit the use of drugs available for protection of neurons (see inhibition of the NMDA receptor).

As POAG drugs like for any drug that needs to be taken (prophylactically) for life, the side effect profile is an important key to ensure patient compliance.

Our understanding of a current POAG drug is that it can be applied topically, reduces IOP, reaches the back of the eye in sufficient concentration to increase optic nerve head perfusion and to protect RGCs.

Two drugs used clinically, brimonidine an α_2_-adrenergic receptor agonist and betaxolol a β-adrenergic receptor antagonist were neuroprotective in animal model and cell culture experiments [44, 112, 128, 132, 152, 194, 197, 198].

Clinical evidence that this neuroprotective effect can also be elicited in human POAG and if so is not coupled to IOP reduction and/or altered perfusion (excluded for brimonidine, 155) [60, 64] remains to be established and would include pharmacodynamic experiments which prove that both drugs, when applied topically, reach the back of the eye in sufficient quantity to be pharmacologically active.

The clinical use of neuroprotective agents e.g. NMDA antagonists will largely depend on advances in pharmacokinetics i.e. if a drug with potentially excellent neuroprotective properties but unacceptable side effects when applied systemically or does not reach the optic nerve head when applied topically can be designed to specifically reach their target cells without interacting with other organs, this drug may be used in the treatment of POAG and would increase our treatment options for a still potentially blinding illness.

Based on the fact that studies in humans proving direct neuroprotection without reducing IOP and / or improve optic nerve head microcirculation will be difficult to justify and the disappointingly slow progress in this field despite the enormous efforts in basic science and the pharmaceutical industry in the field of neuroprotection, for the near future, neuroprotective drugs for glaucoma therapy will most probably exert their effect in a combination of indirect and direct effects i.e. a reduction in IOP and / or improvement of optic nerve head microcirculation and direct neuroprotective action (proven in animal model and cell culture experiments). These drugs will be topically active and need to be demonstrated that they reach the optic nerve head in sufficient concentration to exert their neuroprotective effect(s).

## Figures and Tables

**Fig. (2a):**
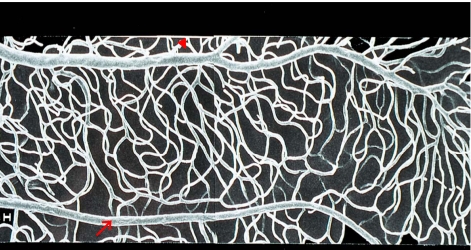
Retinal microvessels (scanning electron micrograph of a vascular resin cast) showing the capillary meshwork between arteriole (arrow) and venule (arrowhead), being tighter on the venular side and wider on the arteriolar side reflecting different oxygen tension in these vessels and thus altered supply.

**Fig. (2b):**
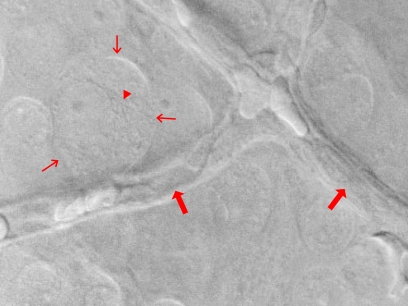
Differential interference contrast microscopy (DIC) of an unstained retina whole mount of a freshly enucleated rat eye. RGCs (thin arrows), even mitochondria are visible: (arrowhead) and capillaries (thick arrows) can be kept well alive up to 9 hours (see 143, 145 and 164).
